# A Rapid, Cost-Effective Pre-Clinical Method to Screen for Pro- or Antiarrhythmic Effects of Substances in an Isolated Heart Preparation

**DOI:** 10.3797/scipharm.1503-03

**Published:** 2015-04-13

**Authors:** John Joseph Borg

**Affiliations:** 1 8, No 40 Dingli Court, Howard Str, Sliema, SLM 1751, Malta; 2 Department of Pharmacology, Medical School, University of Bristol, BS81TD, United Kingdom

**Keywords:** Arrhythmia, Contractile variation, Coronary ligation, Lambeth Conventions, ICHS7B

## Abstract

This study describes a rapid, cost-effective pre-clinical method to screen for pro- or antiarrhythmic effects of substances in an isolated heart preparation in line with the regulatory requirements of ICHS7B. The computational method “MFC method” quantifies arrhythmic episodes from isolated perfused hearts based on measuring the variation in the maximum force of contraction. Experiments were performed on hearts isolated from male Wistar rats. Arrhythmias were induced by the addition of tefluthrin or by ligation of the left coronary artery. The “MFC method” accurately measures the maximum force of every myocardial contraction and correlates it with the magnitude of the preceding beat. Arrhythmias were quantified by determining the coefficient of variation in the maximum force of contraction. This method is a useful approach to quickly identify the pro- or antiarrhythmic effects of drugs prior to more detailed analysis; particularly where the effects are varied and not easily classified under the Lambeth Conventions. Therefore, the “MFC method” can be used as a rapid screen for the antiarrhythmic effects of novel compounds or for rapidly determining potential cardiac toxicity.

## Introduction

To be placed on the market in the European Union (EU), a medicinal product needs a marketing authorisation. To obtain a marketing authorisation, an application consisting of a dossier supporting the medicinal product’s quality, safety, and efficacy needs to be submitted to regulatory authorities. All medicinal products need to fulfil the legislative requirements of Directive 2001/83/EC by showing that the medicinal product to be placed on the market is safe and efficacious (as well as of good quality). Apart from numerous other requirements (see www.ema.europa.eu regulatory guidelines), all medicinal products need to be tested for cardiac safety both pre-clinically and clinically. The regulatory guidelines, International Conference on Harmonisation (ICH) S7A, S7B, and E14 (see www.ema.europa.eu), together lay out the requirements that are needed to establish and verify if active substances induce cardiac toxicity (including arrythmogenesis). Together the regulatory guidelines introduce a tiered approach on establishing the safety of medicinal products through pharmacological and pharmacodynamic studies for human pharmaceuticals. With respect to cardiac safety: ICHS7A requires core battery safety pharmacology studies to be carried out on vital organs or systems (including the cardiovascular system). This includes testing the active substances’ effects on blood pressure, heart rate, electrocardiogram (ECG), methods of repolarization, conductance, and abnormalities through *in vivo*, *in vitro*, and *ex vivo* evaluations. Follow-up safety studies (measuring cardiac output, ventricular contractility, vascular resistance) should be considered to be carried out based on the results obtained from the core battery studies. ICH S7B describes the non-clinical testing strategy for assessing the potential of a test substance and its metabolites to delay ventricular repolarization. The testing strategy would include:

(1) *in vitro* electrophysiology studies that explore the potential cellular mechanism that might not be evident in *in vivo* data. Studies on action potentials resulting in information regarding the integrated activity of multiple ion channels in the heart;
(2) *in vivo* ECG evaluations providing information on conduction properties and non-cardiac influences;
(3) proarrhythmic effects – measured in isolated cardiac preparations or animals;
(4) *in vivo* studies allowing evaluation of the parent substance and metabolites to enable estimation of safety margins.

The guideline also contains decision trees to warrant further investigations at the cardiac level (electrophysiology testing on *human Ether-à-go-go Related Gene* (hERG) channels as well as QT studies) in animal models or in humans. ICH E14 specifies the need for a thorough QT/QTc study or the absence of a need based on the pre-clinical and clinical data available. The ‘thorough QT/QTc’ study should be conducted early in clinical development to determine whether the effect of a drug on cardiac repolarization should be studied intensively in later development stages. All studies carried out must be Good Laboratory Practice (GLP)-compliant. Taken together, the regulatory requirements are clear in showing that there is a need for a rapid and cheap cost-effective method to evaluate if a compound induces cardiac toxicity or not to warrant further development. This paper describes a straightforward method to allow researchers/drug developers to answer the question whether a chemical is pro- or antiarrhythmic. The method is based upon the relationship between the force-interval and arrhythmogenesis [1–5]. Difficulties in the classification, quantification, and analysis of arrhythmias are well-known, although conventions (such as the Lambeth) tried to address these issues by standardising the interpretation of electrocardiograms in a model-independent manner. Distinction between one type of arrhythmia and another is usually based on subjective criteria. In addition, different classifications of arrhythmias have been proposed [6–9]. The exercise of qualifying and quantifying arrhythmias from ECGs in clinical trials is not standardised and false negative results have sometimes been documented [10]. Therefore, a quick and straightforward low-cost method for the non-clinical evaluation of antiarrhythmic (or proarrhythmic) effects of drugs, which can quantify all arrhythmias that involve contractile variability, is lacking. In this paper, such a method for quantifying arrhythmias in the retrograde perfused isolated heart is proposed.

A straightforward method was developed specifically for the rapid identification of antiarrhythmic or proarrhythmic effects of compounds, prior to more detailed analysis. It records the maximum myocardial force of contraction (MFC) of every cardiac cycle and relates it to the next, thereby providing an indication of its regularity. The MFC is a useful parameter to measure since it varies during arrhythmic episodes: a phenomenon first shown by Capogrossi *et al.*, [2]. The “MFC method” represents an excellent cost-effective way of quantifying potential pro- or antiarrhythmic effects of drugs, particularly if this technique is implemented together with ECG/epicardial electrogram (EGM) analysis by published techniques [11–16]. This paper describes the assumptions behind the “MFC method” and evidence for its validity.

## Results

### Control Force of Contraction (FOC)

**Fig. 1:**
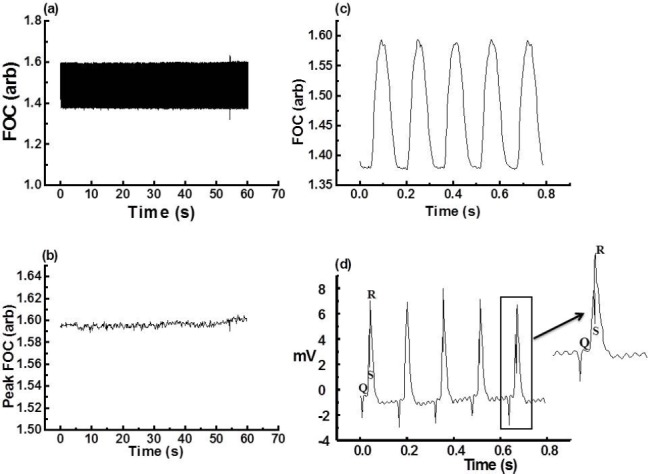
(a) Raw data of a 1-minute FOC control period as acquired by Chart v3.4.8.1^®^, (b) MFC of the raw data shown in (a) as analysed by the “MFC method,” (c) a 0.8-second insert zoom of the raw data shown in (a), (d) corresponding EGM of (c)

The ability of the program to find the number of peaks for different control periods was assessed from recordings of varying time duration. The program’s accuracy in determining the MFC during the control periods was found to be 99% ± 0.02% (n=40). Figure 1 shows the FOC trace recorded (a) and the corresponding MFC calculated by the “MFC method” for a control period of 1 minute (b). Panel (c) and panel (d) show a <1 second recording of FOC with corresponding EGM recordings. Analysis of 22 isolated hearts revealed that for a 10-minute control period (which contained more than 2,000 contractions) the mean CV of the MFC was 0.72 ± 0.066.

### Ability of the “MFC Method” to Determine the Peak FOC During Arrhythmias

**Fig. 2:**
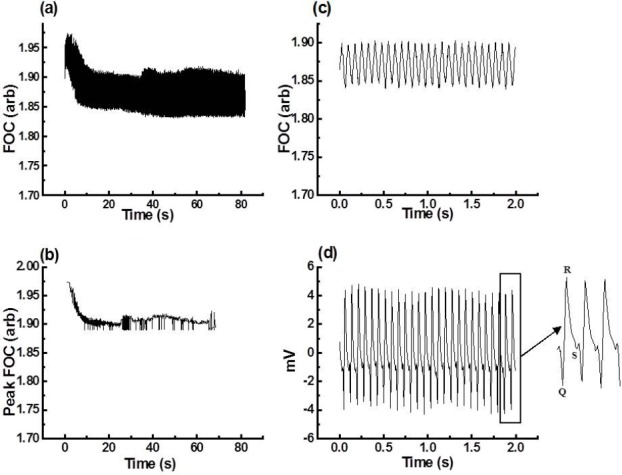
(a) Raw data of a 1-minute 20-second period of VT induced by tefluthrin 10 μM solution, (b) MFC of the raw data shown in (a) as analysed by the “MFC method,” (c) a 2-second insert zoom of the raw data shown in (a), (d) corresponding EGM of (c)

**Fig. 3:**
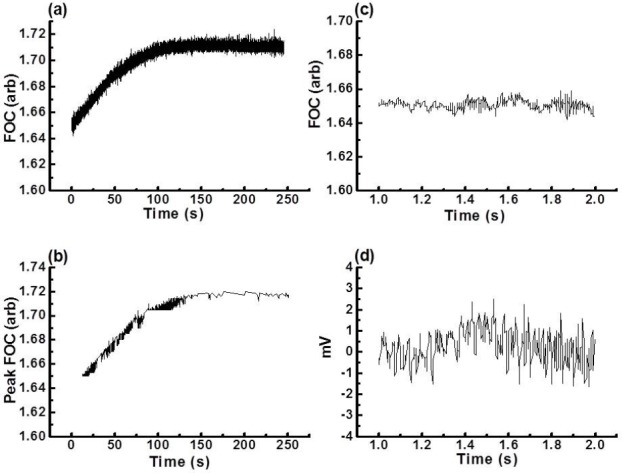
(a) Raw data of a 4-minute 10-second period of VF induced by ligation of the left coronary arteries in the presence of tefluthrin 10 μM, (b) MFC of the raw data shown in (a) as analysed by the “MFC method,” (c) a 1-second insert zoom of the raw data shown in (a), (d) corresponding EGM of (c), note that there is no distinguishable QRS complex in VF

**Fig. 4:**
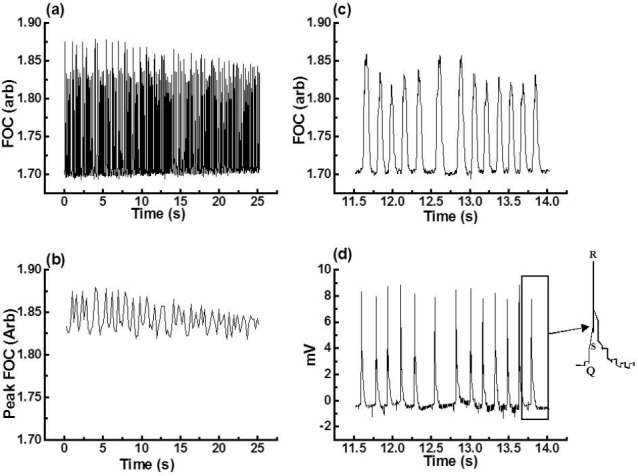
(a) Raw data of a 25-second period of Salvos induced by tefluthrin 10 μM, (b) MFC of the raw data shown in (a) as analysed by the “MFC method,” (c) a 2.5-second insert zoom of the raw data shown in (a), (d) corresponding EGM of (c)

**Fig. 5:**
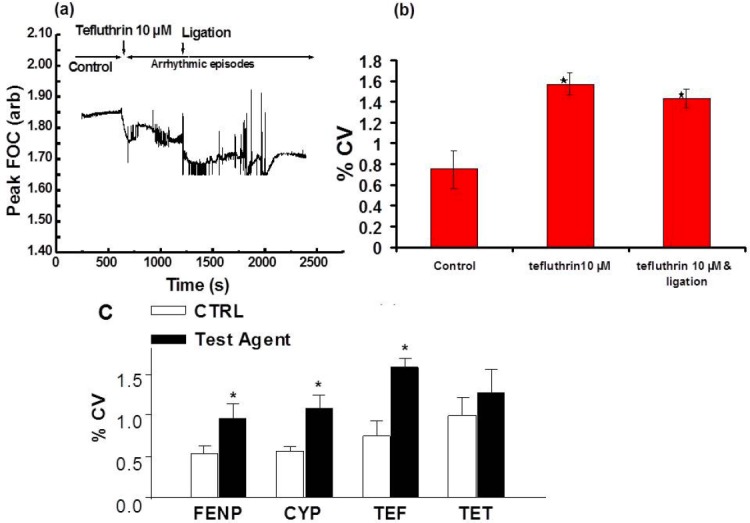
(a) MFCs calculated over 40 minutes by the “MFC method”, showing a control period of 10 minutes and a mixed arrhythmic period of 30 minutes induced by tefluthrin 10 μM and ligation of the left coronary arteries, (b) percentage CV of the MFC of the control (10 minutes), tefluthrin 10 μM (10 minutes) and ligation of the left coronary arteries in the presence of tefluthrin 10 μM (20 minutes) of 5 isolated hearts (n=5). The results show that arrhythmic periods increase the % CV of the MFC significantly (P<0.05), although there is no significant difference in the % CV following coronary artery ligation in the presence of tefluthrin 10 μM. Also shown in (a) is a period of ventricular fibrillation (VF), (c) bar graphs showing the percentage mean coefficient of variation (CV) in MFC during the control and pyrethroid perfusions for each of the compounds indicated below the horizontal axis. No significant difference was observed between CTRL periods prior to pyrethroid perfusion (P>0.05; ANOVA). * denotes statistical significance at P<0.05 as compared to the control. FENP = Fenpopathrin, CYP = Cypermethrin, TET = Tetramethrin

To demonstrate how the “MFC method” analyses different types of arrhythmic episodes, 0.5- to 5-minute periods of Ventricular Tachycardia (VT), Ventricular Fibrillation (VF), and Salvos (identified according to the Lambeth Conventions [9]; following EGM analysis) were subjected to “MFC method” analysis. Figures 2–4 present the FOC traces recorded (panel a) and the corresponding MFCs (panel b) calculated using the “MFC method” for different periods of time for VT, VF, and Salvos. The panels (c) and (d) in Figures 3–5 show an FOC period of less than 3 seconds and their corresponding EGM recordings. These results show that the “MFC method” calculates accurately the MFC of the most common arrhythmic episodes. However, the program’s performance in locating the MFC becomes less accurate when the troughs of the FOC traces recorded by Chart v3.4.8.1^®^ vary by more than 5% (data not shown). It was also observed that the program had difficulty in finding the MFCs of VF because during VF, the FOC decreases to a minimum (also a feature heart block). This, however, is not a limitation of the “MFC method” since in the presence of VF or heart block delta (Δ), the FOC is zero.

The arrhythmias discussed above (which were induced by the application of both tefluthrin 10 µM solution and/or ligation of the left coronary artery) have been selected from FOC and EGM recordings from five hearts. Figure 5(a) presents the MFC (over 40 minutes) for one heart with a control period (10 minutes) and an arrhythmic period following ligation of the left coronary artery after 10 minutes perfusion with 10 µM tefluthrin solution. The arrhythmic period in Figure 5a shows the three types of arrhythmias characterised above.

Note the MFC can also be analysed and presented in terms of its coefficient of variation (CV; Figure 5b) derived from the MFC. The CV can be regarded as a measure of the degree of arrhythmicity: the greater the value for the CV, the more arrhythmic the heart. Thus, in the example presented in Figure 5b, the % CV of the MFC of the control period (0.75 ± 0.18%; n=5) increased to 1.57 ± 0.1% (n=5) in the presence of tefluthrin 10 μM and 1.43 ± 0.09% (n=5) with ligation of the left coronary arteries in the presence of 10 μM tefluthrin. Experiments carried out on isolated hearts demonstrate that pyrethroid agents are proarrhythmic Figure 5c. The results show that arrhythmic periods increase the CV of the MFC significantly (*P*<0.05).

## Discussion

Other investigators have studied the relationship between arrhythmic episodes and the FOC of the heart [5], yet a review of literature in the public domain shows that such a method is not being used to study whether an investigational medicinal substance is arrythmogenic or not. The present study describes a technique for use as a quick and reliable method to quantify arrhythmias on the basis of variation occurring in the FOC during arrhythmic episodes. Such data allow the determination of arrhythmias occurring when test solutions are applied. The coefficient of variation (CV) was used as a parameter for monitoring arrhythmia because it is a straightforward statistical measure of the scatter of variation of data around the mean. Thus, using the CV makes it straightforward to determine whether or not there is an increase in the variation of the MFC and consequently, an increase in arrhythmia. The % CVs for each period (control and test solution) were calculated with n>2000 contractions thus making the % CV a reliable method for determining alterations in the FOC produced by test compounds. Factors that alter force without disturbing the regularity of rhythm (e.g. β-agonists) might alter the arrhythmogenic assessment if a small sample (in terms of time) is taken, but will have no significance on the % CV if a reasonable sampling interval (minutes rather than seconds) is used. The increase in MFC appears to result from an underlying arrhythmogenic dispersion of beat intervals and its influence on the force-interval relationship of the heart. Such variability in inter-beat intervals may be a precursor to serious cardiac arrhythmias such as torsades de pointes or even ventricular fibrillation. Reported mammalian pyrethroid toxicity generally tends to be associated with neuronal rather than cardiac symptoms, and may thus reflect differences in mammalian pyrethroid toxicity due to the route of exposure. It is well-known that the bioavailability for oral administration of pyrethroid agents has been associated with a bioavailability of 0.1 [20]. However, these experiments also demonstrate that cardiac tissue does not show any inherent resistance to pyrethroid toxicity.

The results obtained in this study illustrate the advantages of the utilisation of the “MFC method” for rapidly quantifying arrhythmias by determining the coefficient of variation in MFC. This method is an ideal approach for the quick identification of pro- or antiarrhythmic effects of drugs prior to more detailed analysis (such as ECG analysis), particularly where the effects are varied and not easily classified.

In addition to determining proarrhythmic drug effects, the “MFC method” may be useful to screen for the antiarrhythmic effects of novel compounds against arrhythmias that lead to contractile variability. Arrhythmias induced by chemical, surgical, or other methods such as ac-coupling and reperfusion may also be commendable to study using this method. It should be noted that the program has been specifically designed to determine coefficients of variation, thereby providing a rapid method of detecting the contractile sequelae of arrhythmias. However, it also calculates other parameters, such as change in contractile force (Δ FOC). This straightforward computational method is a robust, reliable method to quickly identify contractile variations in the force of contraction induced by compounds. Thus, the “MFC method” is very useful in situations where the classification and quantification of arrhythmias (based upon the investigator’s subjective criteria to distinguish between one type of arrhythmia and another) is complex and therefore difficult to perform using other methods of analysis.

### Experimental Conditions for Consideration in Using the “MFC Method”

Variation in the baseline level of the troughs of the raw data acquired for the FOC decrease the accuracy with which the “MFC method” calculates the MFC. Such variation occurs by two means: physiological (e.g. direct tissue damage) or disturbances of the experimental setup (e.g. mechanical movement of the force transducer amplifier). Thus, data acquired with a changing ’baseline’ should be discarded. However, careful setting up of the preparation prior to recording in our experience eliminates all these problems.

## Experimental

### Isolated Perfused Rat Hearts

Male Wistar rats (250–300 g; Harlan, UK) were killed by an overdose (200 mg·kg^−1^) of intraperitoneal Euthatal^®^ (pentobarbitone sodium BP; Rhone Merieux, Ireland); a Home Office-approved procedure (Schedule 1) (i.e. UK National Ethical Standards for the Care and Use of Laboratory Animals (University of Bristol)).

The rat was positioned with the ventral surface facing upwards and a thoractomy performed. The heart was rapidly excised and immersed in cold (~4°C) perfusion fluid containing 2.5 mM Ca^2+^. Placing the heart in a cold solution acted to reduce the formation of blood clots and to slow metabolism while the aorta was being tied to the cannula of the Langendorff perfusion apparatus. The heart was then perfused in a retrograde fashion (constant rate of 10 ml·min^−1^ to 11 ml·min^−1^, and 38°C) with a modified Krebs-Henseleit solution (solution 1, see below). As myocytes are acutely sensitive to impurities in the chemical salts used to make up solutions, all solutions used were made from deionised water (Purite Select Analyst HP, Purite Ltd., Oxford) and AristaR grade chemicals (BDH), which are low in impurities. Solution 1 contained (in mM) 118.5 NaCl, 25.0 NaHCO_3_, 1.2 MgSO_4_, 1.2 KH_2_PO_4_, 3.0 KCl, 2.5 CaCl_2_, and 11.1 glucose. Perfusate was equilibrated with 95% O_2_-5% CO_2_ at 38°C, giving a pH of 7.4 resulting in a PO2 of ≥ 550 mmHg, and a PCO2 of 35 mmHg, at constant pressure (80 cm water). Pyrethroids (tefluthrin, cypermethrin, fenpopathrin, & tetramethrin) were purchased from Sigma Chemical Company, Poole, UK. Arrhythmias were induced: (a) by the addition of 10 μM pyrethroids (known to induce arrhythmia in this preparation [18]; from a 10 mM stock solution in DMSO) to the modified Krebs-Henseleit solution or (b) by ligation of the left coronary artery. Figure legends for the data in the ’Results’ section indicate which method(s) was/were employed for particular experiments. Coronary arteries were ligated using a traction-type coronary occluder according to Heimberger [17]. Sham ligations were performed by leaving the ligation loose.

To record the EGM, stainless steel electrodes were placed on the apex of the right ventricle, and under the right atrium. This electrode arrangement gave a clear P wave and a ventricular complex. Separate components of the tripolar EGM were confirmed by individually disconnecting the electrodes to the heart and observing that the negative deflection (P wave) and the positive recording (QRS complex) corresponded to the atrium and ventricle, respectively. The upward QRS complex was also used to calculate the (ventricular) heart rate. Attempts to calculate heart rate from the P wave (atrial rate) were not reliable due to the relatively small size of the P-wave signal, which is typical of rat ECG recordings [18, 19]. Electrical noise was minimised through the use of a ground electrode in the perfusate stream. Cardiac contractile force was measured with a force transducer attached to the ventricular apex by silk thread. The interval between the successive contractions recorded was used to calculate the heart rate. The EGM and FOC were acquired using a PC computer in conjunction with a data acquisition system PowerLab/800^®^ (ADInstruments, California, USA). Data were continuously recorded using Chart v3.4.8.1^®^ (ADInstruments) software at a sampling rate of 400 Hz. These recorded traces were acquired by a multi-channel amplifier (PowerLab/800^®^) and filtered with a low noise channel selector (cutoff frequency at l00 Hz (−3 dB)). Myocardial force of contraction (MFC) was recorded offline from saved text files generated by Chart v3.4.8.1^®^ by an in-house software programme (the “MFC method” see Figure 1). The “MFC method” records the MFC of every cardiac cycle and relates it to the next, thereby providing an indication of the cardiac cycle’s regularity. The MFC is a useful parameter to measure since it varies during arrhythmic episodes.

**Fig. 6:**
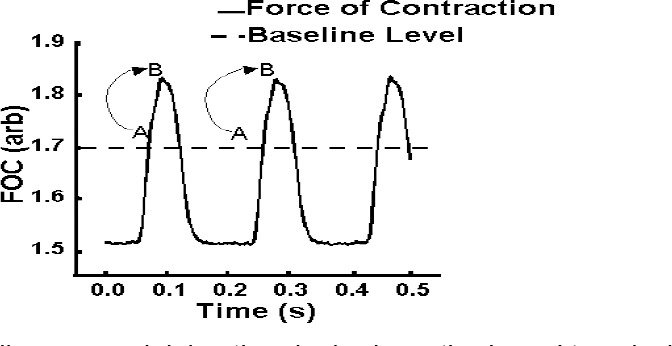
Schematic diagram explaining the algebraic method used to calculate the MFC by the “MFC method”. The Force of Contraction (FOC) trace is the FOC recorded by Chart v3.4.8.1^®^. A is the first datum above the inputted ’baseline’ in the program, while B is the maximum datum on the ascending FOC trace above the ’baseline’

### System Description and Analysis

**Fig. 7:**
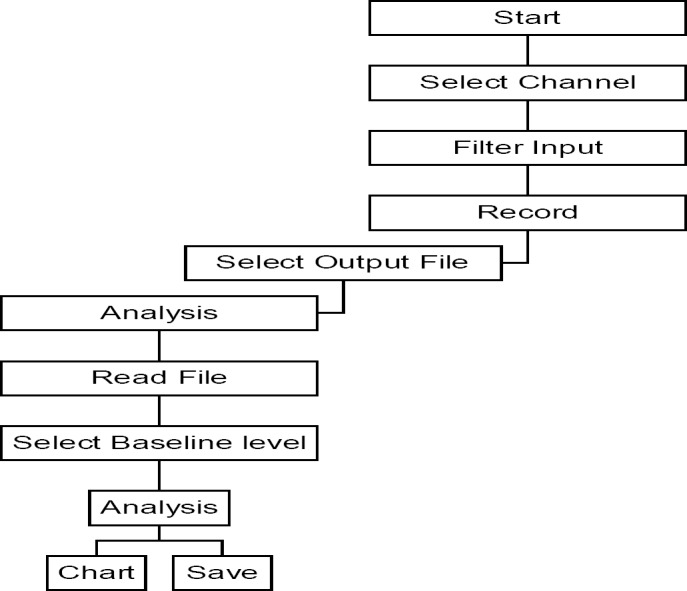
Flow chart of the system. The model setup involves two processes: the acquisition of data and the analysis of the raw FOC data. The steps involved in the analysis of the FOC data can be summarised as: (a) data loading, (b) ‘baseline’ level inputting, and (c) analysis

A flow chart describing the key components of the “MFC method” is shown in Figure 7.

The model setup involves two processes: the acquisition of data and the analysis of raw FOC data. The MFC during the control and arrhythmic periods were calculated offline using the “MFC method” from a saved text version of the FOC trace following settings of the ’baseline’ (see ‘Supporting Information’ 1 for the source code). The “MFC method” then analysed the data points of the text file and recorded the MFC of every FOC cycle by the following algebraic sequence: thus A was defined as the first datum point above the inputted ’baseline’ of the recorded trace (see Figures 1a and b), while B was identified by the program as the MFC above the ’baseline’. Then if A < B, B was saved. Then A was reset as the first datum point above the ’baseline’ on the next ascending FOC trace. Thus, by setting a ’baseline’ depending on the individual traces recorded, the program analysed and recorded peaks over a ’baseline’ selected by the investigator. The ’baseline’ is a user-defined parameter, which only allows data to pass if it is greater in magnitude. The ’baseline’ was set in order to minimise any potential noise and effects of interference occurring during the refractory period of the cardiac action potential. The MFC of the FOC were saved as a text file and plotted using Microsoft Excel^®^. The “MFC method” also calculates the troughs of the FOC trace by the inverse procedure described above and the change in contractile force (Δ FOC) of each beat. Note that during heart block or ventricular fibrillation (VF), the Δ FOC is zero or close to zero.

### Statistical Analysis

The data obtained by the “MFC method” from the analysis of the raw data were then analysed using Microsoft Excel^®^ and the coefficient of variation (CV) was calculated. The CV is a measure of the degree of scatter of data around the mean and is defined as the ratio of the standard deviation to the mean. The closer the CV is to zero, the less scatter of data around the mean is observed. The CV can be regarded as a measure of the degree of arrhythmicity: the greater the value for the CV the more arrhythmic the heart. A paired Student’s t-test was used to calculate the statistical significance between the control and drug periods for the mean CV of the FOC.

## Supporting Information

The source code for the “MFC method” (this program has been written in Delphi programming language and is Windows-compatible) is available in the online version (Type: PDF, Size: < 0.1 MB): http://dx.doi.org/10.3797/scipharm.1503-03

## Author’s Statements

### Disclaimer

The views expressed in this article are the personal views of the author and may not be used or quoted as being made on behalf of, or reflecting the position of, any national competent authority, the EMA or one of its committees, working parties, or any University.
